# The results of a close follow-up of trainees to gain a good blood collection practice

**DOI:** 10.2478/jomb-2019-0053

**Published:** 2020-09-02

**Authors:** Güzin Aykal, Hatice Esen, Ayşenur Yeğin, Cemile Öz

**Affiliations:** 1 Antalya Education and Research Hospital, Clinical Biochemistry, Antalya, Turkey; 2 Antalya Education and Research Hospital, Department of Research and Development, Antalya, Turkey

**Keywords:** phlebotomy, venipuncture education, preanalytical phase, close follow-up, patient safety, flebotomija, venepunktura, preanalitička faza, pažljivo praćenje, bezbednost pacijenata

## Abstract

**Background:**

Phlebotomy is one of the most important steps in the preanalytical phase of a clinical laboratory process. In order to decrease phlebotomy errors, this specific procedure should be taught in detail by laboratory organizations. Our study aims to practice the training program on venous blood sampling and observe the close follow-up results.

**Methods:**

In this observational study, 127 students who started their summer internship in Antalya Education and Research Hospital were given a one-day theoretical phlebotomy training in accordance with the Venous Blood Sampling Guidelines. After the theoretical training, phlebotomy applications of 10 students who were working in the field of out-patient blood sampling were observed both with and without their knowledge. A comprehensive checklist related to phlebotomy was created by the trainers in Antalya Education and Research Hospital and the observers answered each question as yes or no. For the statistical analysis, IBM SPSS Statistics 21.0 was used.

**Results:**

After the theoretical education, the trainees were observed but no significant difference was found between the first and the second informed observations (p = 0.125). The students were observed three times more in the following week without their knowledge. There was a statistically significant difference between the first and the third unannounced observations (p=0.001).

**Conclusions:**

In order to perform phlebotomy correctly, apart from theoretical education, a close follow-up is necessary too.

## Abstract (sr)

**Uvod:**

Flebotomija je jedan od najvažnijih koraka u preanalitičkoj fazi kliničkog laboratorijskog procesa. Da bi se smanjile greške u flebotomiji, laboratorije treba da pruže detaljnu obuku iz ovog specifičnog postupka. Cilj naše studije je sprovođenje programa obuke o uzimanju uzoraka venske krvi i posmatranje rezultata praćenja polaznika te obuke.

**Metode:**

Ova opservaciona studija je uključila 127 studenata koji su započeli letnje stažiranje u Obrazovno-istraživačkoj bolnici u Antaliji. Navedeni studenti su prošli jednodnevno teorijsko usavršavanje iz flebotomije, a u skladu sa Smernicama za uzimanje uzoraka venske krvi. Nakon teorijske obuke, sprovedeno je praćenje primene procedura uzimanja uzoraka krvi kod 10 studenata koji su uzimali uzorke krvi u dnevnoj bolnici, i to sa ili bez njihovog znanja o tome. Treneri u Obrazovno-istraživačkoj bolnici u Antaliji su sačinili sveobuhvatnu listu pitanja, a studenti su na svako pitanje odgovorili sa da ili ne. Za statističku analizu korišćen je program IBM SPSS Statistics 21.0.

**Rezultati:**

Nakon teorijske nastave, sprovedena je opservacija polaznika, ali nije uočena značajna razlika između prvog i drugog puta kada su studenti obavešteni da se vrši opservacija njihovih postupaka (p = 0,125). Sledeće nedelje je provera sprovedena tri puta i to bez znanja studenata. Utvrđena je statistički značajna razlika (p = 0,001) između prvog i trećeg nenajavljenog posmatranja.

**Zaključak:**

Za pravilno uzimanje uzoraka krvi, osim teorijskog obrazovanja, neophodno je i pažljivo praćenje primene naučenog.

## Introduction

Venous blood sampling is one of the most important steps of the preanalytical phase and is the most common invasive procedure in health care. Every step it consists of has a potential risk for patient safety [1] [2] [3]. A large cumulative effect can be created by small variations in each step of phlebotomy. Furthermore, an additional layer of variability is introduced into the system with a very heterogeneous group of medical staff (laboratory technicians, nurses, etc.) that should be educated to perform phlebotomy [4] [5].

Phlebotomy is a composite process that needs accuracy, ability, responsibility and good interaction between the patient and the phlebotomist. It also demands manual skills and theoretical knowledge given in the guideline [6]. To obtain high-quality laboratory results, it is essential for a phlebotomist to be aware of the latest laboratory sampling procedures and to be able to use them in practice. Therefore, the personnel in charge of venipuncture should be well educated and trained to fulfil all these qualifications [7] [8].

Moreover, phlebotomy errors can often go unrecognized, since they are latent and distant from direct control [7]. Consequently, in order to minimize the possibility of errors, this procedure should be taught well by the laboratory organization and internalized by the one who was educated. Therefore, the objective of this study is to observe the results of a close follow-up besides the education program on venous blood sampling applied to the trainee students to gain good blood collection practice.

## Materials and Methods

### Survey Design

This observational survey study was performed in July 2017. A group of 127 students who started summer internships in Antalya Education and Research Hospital (AERH) took part in an education program about phlebotomy in accordance with the Venous Blood Sampling Guideline. After the education program was completed, 10 of the students practising in the out-patient blood sampling area were included for the close follow-up part of the study. All the students who were educated (n=127) came from different health colleges of Antalya, and although legislation of colleges included guidelines for venous blood sampling, compliance training was provided when they came to our hospital.

### The theoretical education programme

All students were invited to a one-day theoretical education programme about venous blood sampling. This programme was planned based on the Venous Blood Sampling Guideline published by the Turkish Biochemical Society [2]. Educators used principles of adult learning theory, and social cognitive theory to help in improving their venipuncture skills. The education program aimed to promote learning and behaviour change by increasing self-efficacy of learners. Main headings were as follows: equipment and supplies, patient preparation, test request form, patient identification, tube labelling, hand sanitization, suitable body areas for blood collection, applying a tourniquet, effective venipuncture technique, order of draw, patient and staff safety, problems and solutions during blood collection, and good communication with the patient.

### Blood collection practice

After the theoretical program, students started their internship in different areas of the hospital. The second step of our study included 10 students practising only in an out-patient blood sampling area. Following the theoretical education, the phlebotomy practice of those 10 students was monitored. For each student, five observations on different days were done while they performed venipuncture.

The first informed observation was carried out during blood collection with the knowledge of the student. A comprehensive checklist containing 10 important steps involved in venipuncture technique was created by educators at AERH (Table 1). The questions in the checklist were answered by the observer as yes or no after each observation. When the blood collection process was completed, the educator asked the student what errors he/she had made in the process and what the deficiencies were during the procedure. The student and the observer reviewed the checklist that the observer had filled out about the venipuncture together. Besides, the students were warned about the mistakes that they were not aware of, and they were told to do the phlebotomy again. The second informed observation was carried out under the same conditions the next day.

**Table 1:** Questions in the phlebotomy performer observation form

Questions		Answers	
		Yes	No
Q1	Did the collector identify the patient according to Venous Blood Sampling Guideline?		
Q2	Did the collector place the tourniquet 4 finger width (10cm) above the venipuncture site?		
Q3	Did the collector clean the venipuncture site correctly?		
Q4	Did the collector leave the venipuncture site untouched post cleaning?		
Q5	Did the collector perform venipuncture properly?		
Q6	Did the collector release the tourniquet when blood flow commenced?		
Q7	Did the collector follow the correct order of draw according to the guidelines?		
Q8	Did the collector perform sampling correct blood volume and ratio of blood to additive?		
Q9	Were all sample tubes immediately and appropriately mixed according to manufacturers specifications?		
Q10	Was the needle/collection system safely and immediately disposed into the sharps container?		

On the third occasion, one week later, they performed blood collection without knowing that they were being observed. After the procedure, the student was informed that he/she had been observed during this process and also was informed about the mistakes they had made. Unannounced observations were repeated two more times on different days until a minimum of errors occurred.

### Statistical analysis

Results are presented as counts and percentages. Differences between observations were analysed with Cochran's Q test. A p-value 0.05 was considered as statistically significant. Different groups were evaluated with a Bonferroni corrected McNemar test [9]. A p-value 0.005 was considered statistically significant. Data were analysed in IBM SPSS Statistics 21.0 (SPSS Inc., Chicago, IL, USA).

## Results

**Figure 1:**
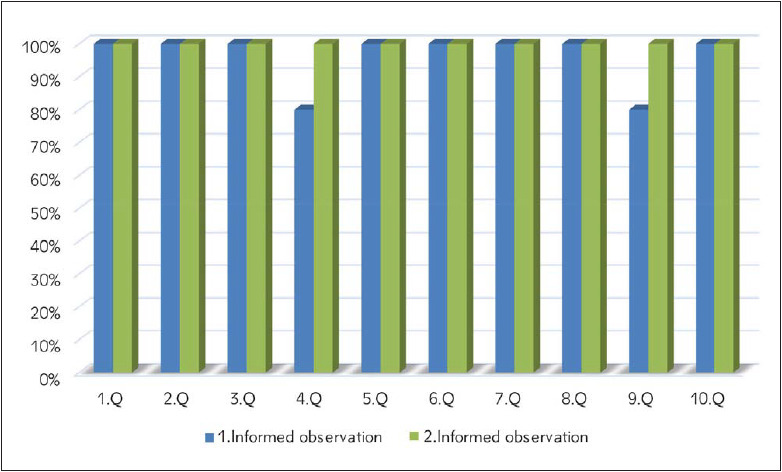
Results of the first and second informed observations (Q: Question)

During the week following the theoretical training, the students were observed for their blood sampling performance. When two consecutive correct venipunctures were performed, the observation was terminated for that day. Ten students who were interns in the outpatient clinic were observed for their venipuncture performance the day after the theoretical education. All students performed nearly correct blood sampling according to the guideline (Figure 1). In the first informed observation, the only mistakes were that two students had repalpation, and the other had not inverted the tubes enough. After each observation, the observer asked the students about his/her mistakes in that process. After being reminded of their mistakes, the students performed a new blood collection while being observed. On the second day of observation, all the students performed the blood collection without any errors (Figure 1).

**Figure 2:**
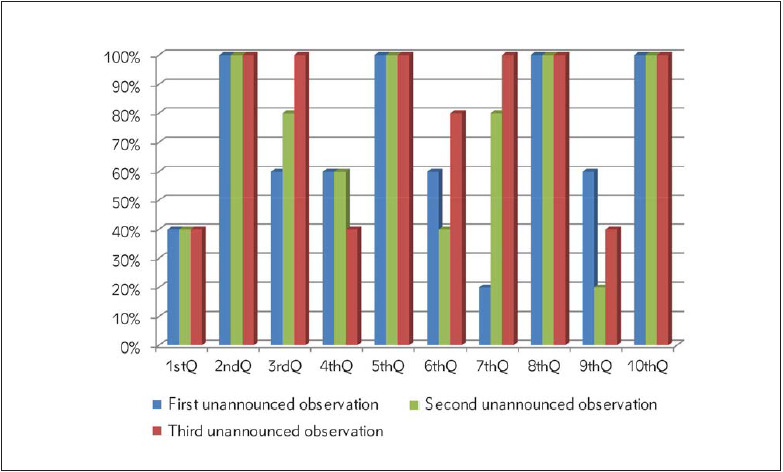
Results of the first, second and third unannounced observations (Q: Question)

In the second phase of our hands-on training, we observed the students without their knowledge in the following week. In the first unannounced observation, only four of the ten students performed correct patient identification, six of ten cleaned the venipuncture site correctly, six of ten students had repalpation, six students removed the tourniquet at the appropriate time, only two of them followed the correct order of draw according to the guidelines, four of ten inverted the tubes for sufficient number of times. After each observation, we described the mistakes they had made in that process. When two consecutive correct venipunctures were performed, the observation was terminated for that day (Figure 2).

During the second unannounced observation, four of ten students identified the patient correctly, eight of ten cleaned the venipuncture site correctly, six of ten students had repalpation, four students removed the tourniquet at the appropriate time, eight students followed the correct order of draw according to the guidelines, and two of ten inverted the tubes for sufficient number of times. All students were immediately notified of their errors (Figure 2).

In the last unannounced observation, six of ten students performed correct patient identification, four students had repalpation, eight students removed the tourniquet at the appropriate time, and only four of ten did perform inversion of the tubes for sufficient number of times (Figure 2).

In all informed and uninformed observations, all the students placed the tourniquet correctly at 4 finger width (10 cm) above the venipuncture site, performed venipuncture properly, performed sampling with correct blood volume, and needles/collection systems were safely and immediately disposed into a sharps container.

There were very few errors in the first informed observation. There were no errors in the second one. Consequently, there was no statistically significant difference between the first and second informed observations (p = 0.125) (Table 2). When all the results were compared, there was a statistically significant difference between the first and the third unannounced observations (p = 0.001) (Table 3). There was a statistically significant difference between the last informed observation and the last unannounced observation (p = 0.001) (Table 4).

**Table 2:** Comparison of the results between informed 1 and informed 2 observations based on McNemar* *McNemar, Q. (1947) Note on the sampling error of the difference between correlated proportions or percentages. Psychometrika 1947; 12: 153–157

Results of the Observations				p
Informed 1	Compliant	Number of Answers	96	0.125
		% within Informed 1	96.0%	
	Noncompliant	Number of Answers	4	
		% within Informed 1	4.0%	
Informed 2	Compliant	Number of Answers	100	
		% within Informed 2	100.0%	
	Noncompliant	Number of Answers	0	
		% within Informed 2	0.00%	

**Table 3:** Comparison of the results between uninformed 1 and uninformed 3 observations based on McNemar* *McNemar, Q. (1947) Note on the sampling error of the difference between correlated proportions or percentages. Psychometrika 1947; 12: 153–157

Results of the Observations				p
Uninformed 1	Compliant	Number of Answers	66	0.001
		% within Uninformed 1	66.0%	
	Noncompliant	Number of Answers	34	
		% within Uninformed 1	34.0%	
Uninformed 3	Compliant	Number of Answers	84	
		% within Uninformed 3	84.0%	
	Noncompliant	Number of Answers	16	
		% within Uninformed 3	16.00%	

**Table 4:** Comparison of the results between informed 2 and uninformed 3 observations based on McNemar* *McNemar, Q. (1947) Note on the sampling error of the difference between correlated proportions or percentages. Psychometrika 1947; 12: 153–157

Results of the Observations				p
Informed 2	Compliant	Number of Answers	100	0.000
		% within Informed 2	100.0%	
	Noncompliant	Number of Answers	0	
		% within Informed 2	0.0%	
Uninformed 3	Compliant	Number of Answers	84	
		% within Uninformed 3	84.0%	
	Noncompliant	Number of Answers	16	
		% within Uninformed 3	16.00%	

## Discussion

We believe this is the first observational study which focused on the change in performance of phlebotomy after a close follow-up of the trainees besides theoretical education. The inspiration for the study came from our experience that students consistently make errors when performing venipuncture in spite of all training, both theoretical and practical.

Laboratory methods change over time, and this can lead to a need to adapt venipuncture procedures and consequently, further training can help to improve practices [5]. The students are required to understand the principles of phlebotomy during their education about the laboratory techniques, but there is not a specialized course about phlebotomy in their routine curriculum [4]. It is thereby essential that lab-oratory medicine recognizes that more focus should be placed on education, training and performance monitoring of phlebotomists [7] [10] [11]. However, convincing the staff to change behaviour to adapt more closely to venipuncture guidelines and other recommended practices has proved to be a challenging task [11] [12]. To achieve improvement in modifying staff behaviour, observational studies are seldom used. In fact, such studies have both the advantage of direct inspection of specimen collection errors and also allow an error frequency determination for each key issue [13].

This study also provides practical strategies for measuring changes in skills and knowledge. Some of these can be used to evaluate the effectiveness of undergraduate and graduate education programs [14]. To assess critical steps in phlebotomy, using a template checklist and risk analysis are efficient methods while observing venous blood specimen collection in practice [13].

Patient safety related to patient identification is a controllable challenge in all types of blood collection procedures [15]. However, in this study, the most challenging steps of the phlebotomy procedure were patient identification and inversion of the tubes for a sufficient number of times. With all the close followup of the trainees, there was no improvement regarding these steps; the same number of students did not identify the patient correctly and an even fewer number of students inverted the tubes for a sufficient number of times.

In our hospital, a phlebotomist uses the IDlabelled tubes the laboratory secretary has already labelled. The students observed in this study should have checked if the tubes belonged to the patient he/she was going to perform the phlebotomy on. Therefore, in this study, patient identification was not a big challenge for a phlebotomist, but they had to confirm that the tubes actually belong to the patient whose blood was taken. In the informed observations, the students were careful about our patient identification procedure, but in the first unannounced observation only 40% of them confirmed that the labelled tubes belonged to the patient. After a close follow-up, on the third unannounced observations again only 60% did the confirmation of the patient identification (Figure 2). Similarly, the EFLM group found in an observational study of venepuncture procedures that the accordance with the CLSI guideline in European countries was extremely low and they observed that patient identification and tube labelling were the most critical steps [13]. Therefore, continuous education of staff for venous blood sampling and monitoring of identification errors have recently been recommended by EFLM [16].

Another important issue in this study was that students would not invert the tubes for sufficient times. Regarding mixing/inverting the test tubes after blood sampling, the majority followed the guidelines; but on the first and the third unannounced observations, there were 40% of students who did proper mixing. On the other hand, the interns were inverting all the tubes but the inverting times for most tubes were below the standard. Likewise, in a previous study in China, the inverting times for most of the tubes were below the standard; therefore, the researches commented that only 22.5% of the nurses were aware of the correct definition of an inversion [17]. Test tube mixing is important after sampling because the collected blood should be mixed properly with the additives in the test tube. Contradictive instructions and unsettled recommendations can adversely affect the phlebotomists' attitudes for change. If personnel believe that following guidelines would result in improved patient outcomes and improved working conditions, they probably would be more likely to change behaviours [18].

The other three steps of phlebotomy that the students had problems with but had improved after a close follow-up training were about repalpation, removing the tourniquet at the appropriate time and following the correct order of draw. In the third unannounced observation, all the students cleaned the venipuncture site properly, but only six of them (60 %) left the venipuncture site untouched after cleaning. Even though they knew that it should be intact, we observed that the students were not self-confident enough and they could not stop themselves from repalpation.

In our study, the correct rate on tourniquet release time was 80 % at the end of the close followup training, 60 % on the first unannounced observation, and 100 % on the informed observation. However, previous studies demonstrated that less than 50% of their phlebotomists did not release the tourniquet when the first test-tube had blood inflows [13] [17]. Moreover, following the correct order of draw step of phlebotomy was perfectly done in informed observations but in the first unannounced observation only 20 % performed it correctly. With a close followup, all ten students followed the correct order of draw according to the guidelines.

The other four steps of phlebotomy observed in our study were well done by the students and all ten of them placed the tourniquet 4 fingers (10 cm) widths above the venipuncture site, performed the venipuncture according to the guidelines, collected the proper amount of blood, and disposed of collection systems immediately (Figure 2).

The difference in the adherence to some of the guidelines practice in phlebotomy may be due to different education of the phlebotomy staff, various settings and relations [16]. As reported in a previous study about the skill development of occupational therapists, behaviour change is difficult. McClusky et al. [14] showed that even the most motivated of health professionals face barriers when attempting to stay up to date by finding, reading and using research. As mentioned before, education and training may improve guidelines adherence, but accreditation of phlebotomy has only marginal effects. In other words, preanalytical conditions should be regularly analysed by the laboratory organizations and the accreditation bodies, in turn, examine the laboratory's accordance with the guidelines [11] [13].

There are some limitations to our study. First of all, the questionnaire for investigation was selfdesigned without undergoing a thorough reliability and validity test. Another limitation was that the long term effects of our close follow-up could not be observed because the students' internship ended in a short time.

As a conclusion of the study, the rate of error was reasonably low in the informed observations of students who undertook a theoretical education on phlebotomy, but this rate increased significantly on the following days where the same students were observed with unannounced observations (Figure 1 and Figure 2). After a close follow-up of three unannounced observations, the trainees were only 80% correct in the venipuncture process. These results suggest that only a theoretical education is not enough for training the personnel for obtaining a guideline-correct phlebotomy procedure, but also a close follow-up is necessary.

*Acknowledgements*. This article is a detailed summary of an oral presentation delivered during the International Biochemistry Congress 2017 /28 the National Biochemistry Congress. Ataturk University Erzurum (Turkey),19–23 September 2017.

## Conflict of interest statement

The authors state that they have no conflicts of interest regarding the publication of this article.
